# Sampling Modification Effects in the Subgingival Microbiome Profile of Healthy Children

**DOI:** 10.3389/fmicb.2016.02142

**Published:** 2017-01-18

**Authors:** Elisabeth Santigli, Slave Trajanoski, Katharina Eberhard, Barbara Klug

**Affiliations:** ^1^Division of Oral Surgery and Orthodontics, Department of Dental Medicine and Oral Health, Medical University of GrazGraz, Austria; ^2^Center for Medical Research, Medical University of GrazGraz, Austria

**Keywords:** oral microbiome, subgingival biofilm, healthy children, next generation sequencing (NGS), 454-pyrosequencing, paper point, subgingival sampling

## Abstract

**Background:** Oral microbiota are considered major players in the development of periodontal diseases. Thorough knowledge of intact subgingival microbiomes is required to elucidate microbial shifts from health to disease.

**Aims:** This comparative study investigated the subgingival microbiome of healthy children, possible inter- and intra-individual effects of modified sampling, and basic comparability of subgingival microprints.

**Methods:** In five 10-year-old children, biofilm was collected from the upper first premolars and first molars using sterilized, UV-treated paper-points inserted into the subgingival sulcus at eight sites. After supragingival cleaning using an electric toothbrush and water, sampling was performed, firstly, excluding (Mode A) and, secondly, including (Mode B) cleansing with sterile cotton pellets. DNA was extracted from the pooled samples, and primers targeting 16S rRNA hypervariable regions V5 and V6 were used for 454-pyrosequencing. Wilcoxon signed rank test and *t*-test were applied to compare sampling modes. Principal coordinate analysis (PCoA) and average agglomerative hierarchical clustering were calculated with unweighted UniFrac distance matrices. Sample grouping was tested with permutational MANOVA (Adonis).

**Results:** Data filtering and quality control yielded 67,218 sequences with an average sequence length of 243bp (SD 6.52; range 231–255). *Actinobacteria* (2.8–24.6%), *Bacteroidetes* (9.2–25.1%), *Proteobacteria* (4.9–50.6%), *Firmicutes* (16.5–57.4%), and *Fusobacteria* (2.2–17.1%) were the five major phyla found in all samples. Differences in microbial abundances between sampling modes were not evident. High sampling numbers are needed to achieve significance for rare bacterial phyla. Samples taken from one individual using different sampling modes were more similar to each other than to other individuals' samples. PCoA and hierarchical clustering showed a grouping of the paired samples. Permutational MANOVA did not reveal sample grouping by sampling modes (*p* = 0.914 by *R*^2^ = 0.09).

**Conclusion:** A slight modification of sampling mode has minor effects corresponding to a natural variability in the microbiome profiles of healthy children. The inter-individual variability in subgingival microprints is greater than intra-individual differences. Statistical analyses of microbial populations should consider this baseline variability and move beyond mere quantification with input from visual analytics. Comparative results are difficult to summarize as methods for studying huge datasets are still evolving. Advanced approaches are needed for sample size calculations in clinical settings.

## Introduction

Oral bacterial biofilm research is an emerging field. During the last decades, the profiling of oral microbial communities has evolved from bacterial culture experiments to biofilm characterization by detailed classification using culture-independent methods (Jenkinson, 2011; Diaz, 2012; Simón-Soro et al., 2013). High throughput next generation sequencing (NGS) like 454-pyrosequencing and metagenome analysis have replaced fingerprinting methods (Ahn et al., 2011; Griffen et al., 2011; Alcaraz et al., 2012; Li et al., 2012, 2013; Siqueira et al., 2012; Abusleme et al., 2013; Trajanoski et al., 2013; Chen et al., 2015; Park et al., 2015). Instead of identifying single bacteria, operational taxonomic units (OTUs) based on sequence similarities (of mostly 97%) are assigned to identify groups of bacteria. This has led to a new research avenue leaving single germ detection behind and looking ahead to a fingerprinting of the whole bacterial community. With this unique microbial fingerprint, even forensic analyses could be made possible, as the composition of bacterial biofilm differs from person to person, whether sampled from the oral cavity (Aas et al., 2005) or the skin (Fierer et al., 2010). The oral microbiome displays a large variability; various microhabitats like gingival tissue, tongue, saliva, supra- or subgingival locations facilitate biofilm formation and growth already at early ages (Papaioannou et al., 2009). Keijser et al. (2008) showed that the vast majority (namely 99.6%) of sequences in saliva and subgingival plaque samples of adults belong to one of the seven major phyla: *Actinobacteria, Bacteroides, Firmicutes, Fusobacteria, Proteobacteria, Spirochetes*, or candidate division TM7. Lazarevic et al. (2010) could prove these findings in salivary samples. However, not only bacterial phyla can be tagged; these new methods can show bacterial diversity on all taxonomic levels from the phylum through the genus level. This identification of bacteria takes place over nine hypervariable regions (V1 through V9) of the 16S rRNA gene used to distinguish thousands of species sequences of one sample from another (Chakravorty et al., 2007; Huse et al., 2008). The huge amount of sequence data gained with these methods puts common knowledge of pathogens into a new perspective. Many bacteria previously known to be pathogens were now also found in healthy subjects. Certain bacterial species like *Streptococci* or *Acinetobacter* were more related to health while other like *Treponema, Fusobacteria*, and *Prevotella* were associated with oral disease states in adults (Ledder et al., 2007; Abusleme et al., 2013; Wade, 2013). At the same time, hundreds of rare bacteria have been neglected in analysis which may be due to their being difficult to cultivate and/or detect, or because their detected numbers do not allow for statistical analysis. Focusing on single species can lead to distortion of the real picture of disease. But, how can we compare patients, possible treatment effects, sampling methods, etc. when the information we get consists not only of 20 bacteria but of thousands of species? In addition, how can clinicians translate this information? In this work, we test and show the exemplary comparison of two subgingival biofilm sampling modes for 454-pyrosequencing. We hypothesize that a modification of the clinical sampling mode can lead to a difference in the microbiome composition. We discuss statistical analyses and bioinformatics to provide information on how to compare on an inter- and intra-individual level the microbiota of the subgingival biofilm of healthy children. Issues related to small sample sizes and sample size calculation are also addressed. The overarching aim of this study is to reach the community of dentists and orthodontists with yet scarce knowledge of the potential of microbiome studies. We wish to raise interdisciplinary awareness for the clinical perspective of oral microbiome research in view of translational medicine from bench-side to patient. According to this announcement we, firstly, address the influence of external factors (i.e., clinical sampling methods) on the stability of a microbiome and, secondly, aim to support methods, possibilities and approaches to change and control the subgingival microbiome in human disease through our clinical work and toward a standardized pipeline. Finally, we look at interdisciplinary collaborations to facilitate the transfer of oral microbiome data to real clinical application.

## Materials and methods

### Subjects

**Figure 1:**
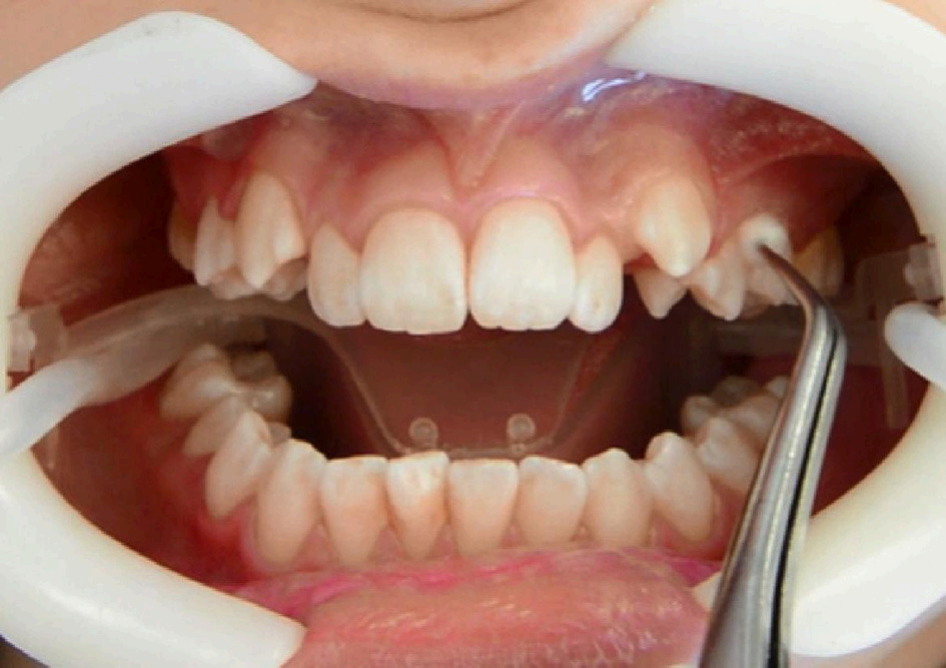
**Sampling in the maxilla after full arch isolation**.

For this comparative study, we included five ten-year-old children of both sexes (two male, three female). All recruited children had fulfilled the following criteria for participation in this study: late mixed dentition with first premolars fully erupted in the upper arch, good general and periodontal health, no bleeding on probing, a plaque index below 30%, no antibiotic intake within the previous 3 months, and no use of antiplaque solutions. Prior to enrollment, written informed consent was obtained from each participant and one of his or her parents. The study was approved by the institutional review board at the Medical University of Graz. Written consent was also obtained explicitly for the publication of the intraoral photo in Figure 1.

### Sampling of subgingival biofilm: modes A and B

**Figure 2:**
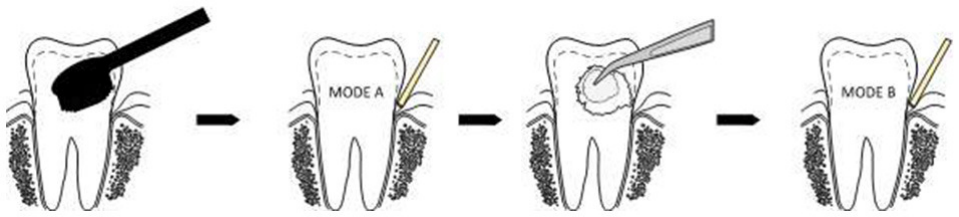
**Subgingival paper point sampling before (Mode A) and after (Mode B) supragingival cleansing with a sterile cotton pellet**.

**Figure 3:**
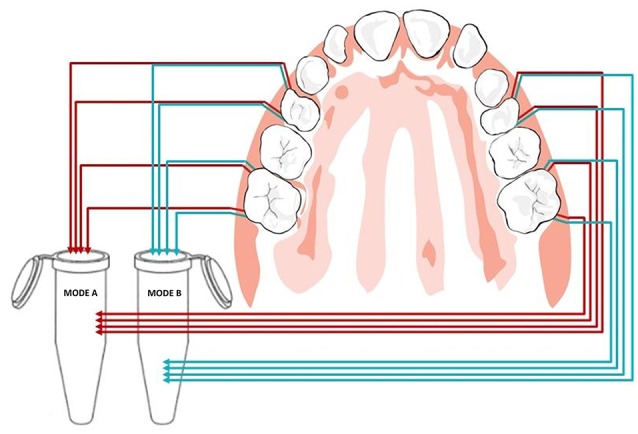
**Pooled paper point sampling of the gingival sulcus: paper points were inserted at eight sites before (Mode A) and after (Mode B) supragingival cleansing shown in red and blue, respectively**.

Clinical examination and sampling was performed by a single, experienced investigator. Sampling was always performed after standardized oral hygiene instructions over a period of 3 weeks. Prior to subgingival sampling, supragingival plaque—as disclosed by an indicator—was removed with a previously unused toothbrush and water performed by the children themselves. No toothpaste was used. Full arch isolation in the maxilla was obtained by NOLA Dry Field © system as shown in Figure 1. Subgingival biofilm was then collected from the upper first premolars and first molars. Biofilm sampling had to be performed very carefully, so as not to traumatize the young gingival tissue in the absence of pockets commonly seen in periodontal disease. Healthy subjects and especially children have small compartments that make probing subgingival biofilm very challenging. Sterilized and UV-treated paper points (ISO15, Antaeos®) were inserted into the subgingival sulcus parallel to the gingival margin at eight sites located mesio- and distobuccally of the four index teeth in two run-throughs differing slightly in their sampling mode. Sampling was done, firstly, excluding (Mode A) and, secondly, including (Mode B) supragingival cleansing with a sterile cotton pellet (see Figure 2). So the main difference between Mode A and Mode B refers to the supragingival cleaning. Samples were taken in sequence during the same sampling procedure from the same eight sites and then pooled and stored at −20°C until processed (see Figure 3).

### DNA extraction

Bacterial DNA was prepared by first placing the paper points in a mixture of 380 μl of MagNA Pure Bacteria Lysis Buffer (Roche Applied Science, Mannheim, Germany) and 20 μl of proteinase K solution (20 g/l). The suspension (including the paper points) was incubated at 65°C for 10 min and subsequently at 95°C for another 10 min. After removal of the paper points, the suspension was transferred into the MagNA Pure Compact Sample Tube (Roche Diagnostics, Mannheim, Germany). Automated DNA extraction was performed on the MagNA Pure Compact instrument (Roche) according to manufacturer instruction using the MagNA Pure Compact Nucleic Acid Isolation Kit I (Roche Diagnostics, Mannheim, Germany). Prior to the start of DNA extraction, the instrument adds the heterologous IC automatically. For extraction of bacterial DNA, the DNA Bacteria Purification protocol was used according to manufacturer instructions. DNA was eluted in 50 μl dH_2_O and stored at −20°C until use.

### 454-pyrosequencing

Pyrosequencing was performed by DNAvision (avenue George Lemaitre 25B, 6041 Charleroi, Belgium, http://www.dnavision.com/). Microbial diversity was analyzed targeting 16S rRNA hypervariable regions V5 and V6. Pyrosequencing primers used are shown in Supplementary Table S1 containing 16s rRNA target specific primer sequences 784F-5′-AGAGTTTGATCCTGGCTC-3′ and 1061R-5′ ATTACCGCGGCTGCTGG-3′ (italic) according to Andersson et al. (2008), MID sequence (underlined), four bases key sequence and the Roche Titanium adaptor sequences (bold). For each sample, a PCR mix of 100 μl was prepared containing 1 × PCR buffer, 2U of KAPA HiFi Hotstart polymerase and dNTPs (Kapa Biosystems), 300 nM primers (Eurogentec, Liege, Belgium), and 60 ng total DNA. Thermal cycling consisted of initial denaturation at 95°C for 5 min, followed by 25 cycles of denaturation at 98°C for 20 s, annealing at 56°C for 40 s, and extension at 72°C for 20 s, with a final extension of 5 min at 72°C. Amplicons were visualized on 1% agarose gels using GelGreen Nucleic Acid gel stain in 1xTAE (Biotium) and were cleaned using the Wizard SV Gel and PCR Clean-up System (Promega, Mannheim, Germany) according to manufacturer instructions. Amplicon DNA concentrations were determined using the Quant-iT PicoGreen dsDNA reagent and kit (Life Tech, Carlsbad, USA) following manufacturer instructions. After quantitation, cleaned amplicons were mixed in equimolar ratios into a single tube. The final pool was again purified using Agencourt Ampure XP purification systems according to manufacturer instructions (Agencourt Biosciences Corporation-Beckman Coulter, USA) and then eluted in 100 μl of 1xTE. The concentration of the purified, pooled DNA was determined using the Quant-iT PicoGreen dsDNA reagent and kit (Life Tech, Carlsbad, USA) following manufacturer instructions. Pyrosequencing of an equimolar pool of 10 samples on 1/8 PTP was carried out using primer A on a 454 Life Sciences Genome Sequencer FLX instrument (Roche, Mannheim, Germany) and following GS FLX Titanium Sequencing Kit XLR70 chemistry (Roche 454 Life Science, Branford, CT, USA) according to manufacturer instructions which resulted in 4131–19,943 raw reads per sample. Sequences are available at NCBI, accession number: SRP080750.

### Sequence data analysis

In the first step, generated sequence data was assessed for quality. By using our own perl script only sequences with a minimum length of 150 bases, average Phred score of 25 and no ambiguous bases were selected for use in the downstream analysis. The remainder of the analysis was performed with the Quantitative Insights Into Microbial Ecology (QIIME) pipeline version 1.3.0 using standard parameters, including uclust (Edgar, 2010) for building OTUs with a similarity threshold of 0.97, pyNAST (Caporaso et al., 2010) for representative sequence alignment, FastTree (Price et al., 2009) to generate the phylogenetic tree and RDP classifier (Wang et al., 2007) for taxonomic assignment. Chimeric sequences were removed using ChimeraSlayer with default QIIME settings after OTU picking and taxonomic assignment on aligned representative sequences.

Estimation of the within-samples diversity (alpha diversity) was performed with the Simpson (1949), Shannon (1948), and Chao (1984) metrics.

In the final step, we generated PCoA plots and performed hierarchical clustering analysis based on distance matrix from an unweighted UniFrac phylogenetic method (Lozupone et al., 2011) which enabled the between-samples comparison (beta diversity) of the microbial communities.

For the beta diversity analysis and normalization, sample heterogeneity was excluded by rarefication of all samples to the sample with the lowest number of reads.

### Statistical analysis

Statistical analyses were performed using SPSS version 22.0 (SPSS Inc., Chicago, IL), R version 3.11 (R Core Team, 2015) and PASS 2012 (NCSS, LLC. Kaysville, Utah). Data are presented as median and as interquartile range (lower quartile 25-percentile and upper quartile 75-percentile). Inter-individual differences of the median relative abundances served for the comparison of the two sampling modes. Wilcoxon tests with Bonferroni correction for multiple testing were used to compare Mode A (excluding supragingival cleansing) and Mode B (including supragingival cleansing) on phylum level (*n* = 6), on class level (*n* = 14), on order level (*n* = 19), on family level (*n* = 27) and on genus level (*n* = 29). All names of the specific *bacteriae* are provided in **Tables 2.1–2.5** and in **Figure 6**. Paired *t*-tests were performed additionally, since the small sample size did not allow to verify the assumption of normality for the data. All reported *p*-values were two-sided. After Bonferroni correction statistical significance was considered with *p* < 0.0083 at the phylum level, *p* < 0.0036 at the class level, *p* < 0.0026 at the order level, *p* < 0.0019 at the family level and *p* < 0.0017 at the genus level.

To test differences in abundance for a total of n taxa between two groups, the rank-sum test including multiple testing with Bonferroni correction was used to estimate the power and the sample size for different effect sizes for alpha level of 0.05/n.

Significance for PCoA (beta-diversity) analyses was checked with multivariate permutation tests using the nonparametric method “Adonis” (999 permutations) included in the package “vegan” of the QIIME-incorporated version of “R.”

## Results

### Pyrosequencing and diversity indices

A total of 92,680 sequences were derived from pooled DNA of 10 samples in the pyrosequencing assay. Data filtering and quality control resulted in 67,218 sequences with an average sequence length of 243 bp (SD 6.52; range 231–255), read numbers per sample ranging from 2937 to 14,629 sequences.

Rarefaction curve analysis showed that the sequencing effort was not sufficient to cover the whole microbiota in the analyzed samples. It is very likely that rare taxa and taxa with low abundances have been missed (Supplementary Figure S1). Nevertheless, this should not significantly influence results, since low abundant taxa do not shift the complete microbiota profiles and the tools used for their comparison are robust enough to compensate for low deviances.

The number of OTUs defined at 97% identity ranged from 532 to 1107 (as shown in Table 1). Sample richness, which in this analysis equals to the number of OTUs, as well as sample diversity (Shannon Index range 4.26–5.31) did not demonstrate major differences between the two sampling modes.

**Table 1:** **Sequencing information and diversity estimates for the subgingival microbiome profiles in five healthy children before (Mode A) and after (Mode B) supragingival cleansing**.

**PatID**	**nReads**	**lAvg**	**nReads after trimming**	**richness (nOTUs)**	**Evenness**	**Shannon**	**Simpson**	**S.chao1**	**se.chao1**	**S.ACE**	**se.ACE**
1A	9628	248.56	7298	758	0.70	4.61	0.96	1359	86.95	1358	22.22
2A	6866	249.21	5086	621	0.72	4.65	0.97	1171	85.29	1259	23.53
3A	4131	239.85	2937	599	0.83	5.31	0.99	1443	138.83	1385	23.33
4A	19,943	241.53	14,629	1099	0.68	4.77	0.97	1827	88.98	1899	26.73
5A	6193	238.53	4425	662	0.79	5.14	0.98	1174	77.59	1239	22.17
			avg	748	0.74	4.90	0.97	1395		1428	
			min	599	0.68	4.61	0.96	1171		1239	
			max	1099	0.83	5.31	0.99	1827		1899	
1B	8817	245.08	6497	887	0.75	5.12	0.98	1592	91.60	1679	25.74
2B	4987	254.8	3861	532	0.72	4.53	0.95	1053	87.21	1162	22.73
3B	11,711	240.16	8348	786	0.64	4.26	0.94	1329	77.56	1376	22.12
4B	14,979	231.59	10,166	1107	0.68	4.75	0.96	1796	81.13	1938	27.41
5B	5425	242.6	3971	546	0.73	4.60	0.97	1042	82.73	1093	20.61
			avg	772	0.70	4.65	0.96	1362		1450	
			min	532	0.64	4.26	0.94	1042		1093	
			max	1107	0.75	5.12	0.98	1796		1938	
**Filtered sequence summary**											
Sequence Alphabet Filter			–								
Primer trimmer			1980								
N count > 0 seq filter			908								
GreaterThan 150 sequence length filter			22,574								
Exponential Quality Filter			–								
Total numbers of OTUs similarity 97%			5601								

### Taxonomic summary

**Figure 4:**
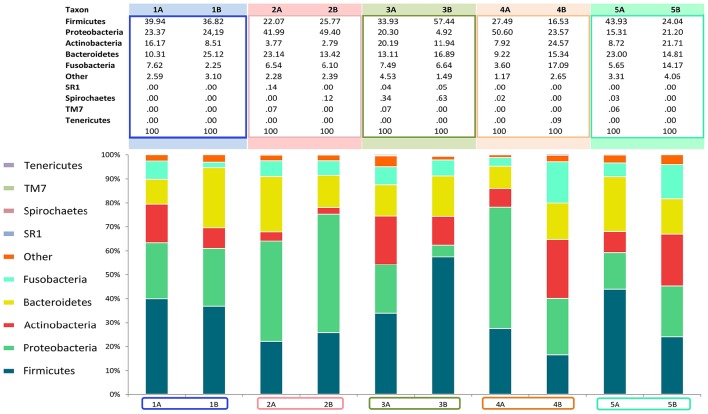
**Relative abundance of all phyla identified in the subgingival microbiome of 5 healthy children before (Mode A) and after (Mode B) supragingival cleansing**.

Analysis over the whole microbiota showed a predomination of the five main phyla: *Actinobacteria* (2.8–24.6%), *Bacteroidetes* (9.2–25.1%), *Proteobacteria* (4.9–50.6%), *Firmicutes* (16.5–57.4%) and *Fusobacteria* (2.2–17.1%) (Figure 4).

The median relative abundances for all representatives in the profiled microbiomes on different taxonomic levels (phylum, class, order, family and genus) are given in Table 2.

**Figure 5:**
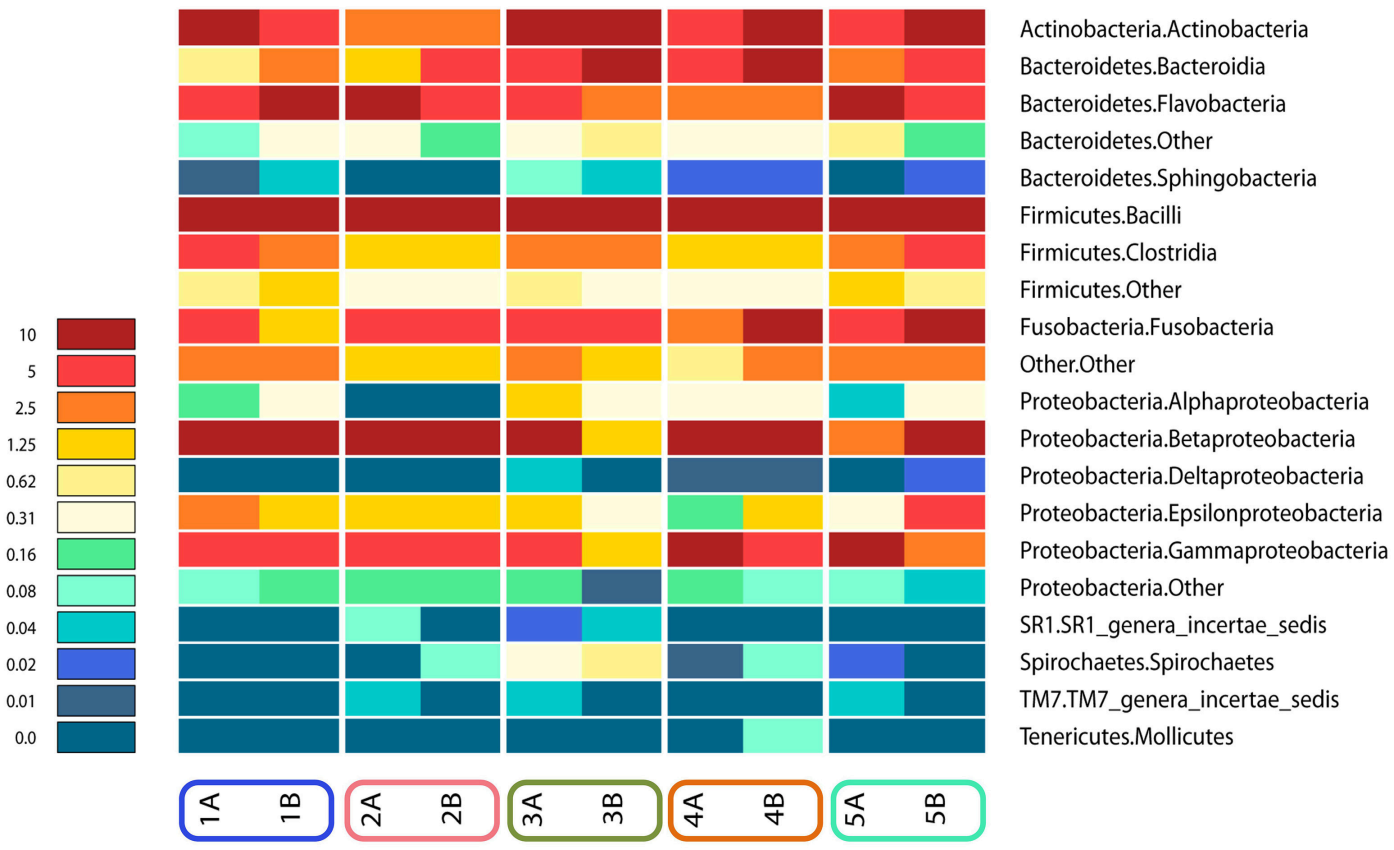
**Heat map of relative abundances of all classes identified in the subgingival microbiome of 5 healthy children before (Mode A) and after (Mode B) supragingival cleansing**.

Figures 4, 5 show sampling modification effects on relative abundances on phylum level (barchart) and on class level (heat map). A concordant qualitative pattern within individuals and differences between individuals could be shown regardless of the sampling mode.

### Statistical analyses of sampling modes and sample size calculation

#### Differences between sampling modes A and B

**Figure 6:**
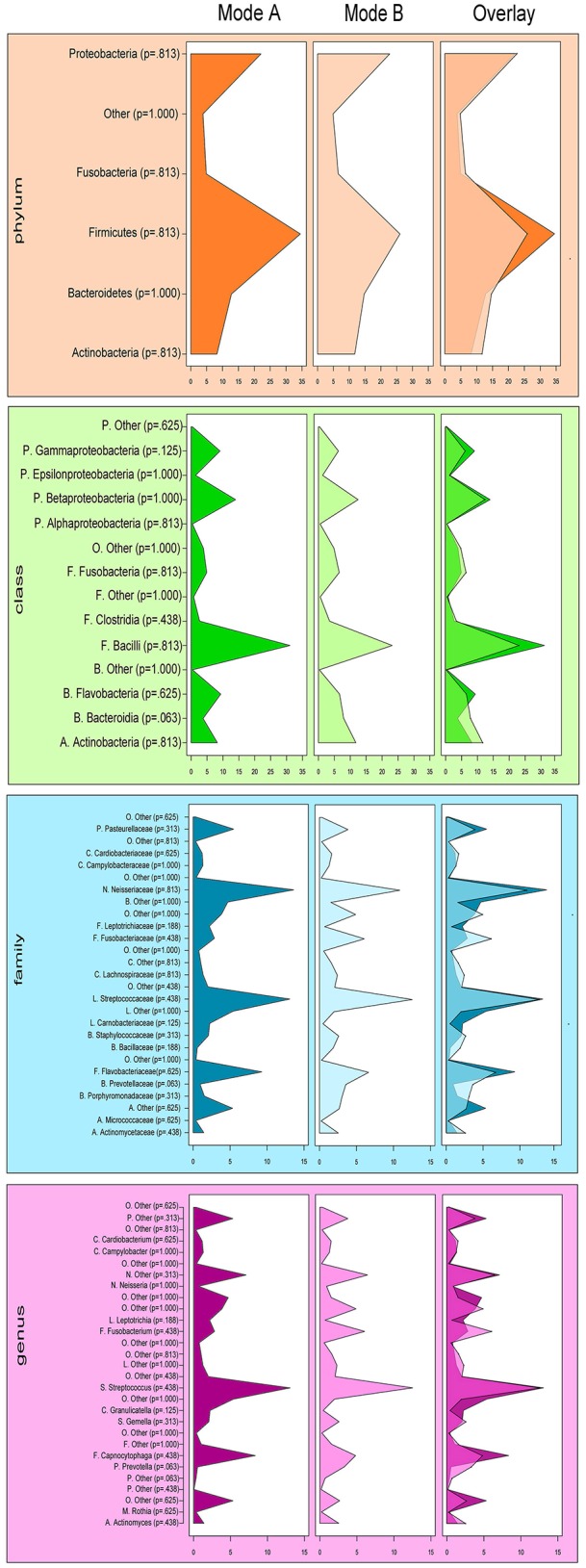
**Comparison of the median relative abundance of bacterial species present in all samples before (Mode A) and after (Mode B) supragingival cleansing (phylum, class, family and genus taxon)**.

Effects in the subgingival microbiome profiles possibly due to sampling modification are displayed by area graphs in Figure 6. *P*-values from Wilcoxon signed rank tests and the median relative abundance at all five taxonomic levels were used to display differences between Mode A (excluding supragingival cleansing) and Mode B (including supragingival cleansing) for all bacterial species on all levels (Tables 2.1–2.5). Nearly statistically significant differences (*p* = 0.063) between sampling Modes A and B could be shown for the phylum of Bacteroidetes based on Wilcoxon signed-rank tests: *Bacteroidia* (class), *Bacteroidales* (order), *Prevotellaceae* (family), and *Prevotella* (genus). The latter was shown to be statistically significant (*p* = 0.047) when the paired *t*-test was applied. Paired *t*-tests were assessed additionally to Wilcoxon signed-rank tests due to the small sample size in the study so as to prove that nearly statistically significant results with Wilcoxon signed-rank tests become significant. In general, the Wilcoxon signed-rank test cannot be significant for a sample size smaller than 6, for two sided testing. For one sided testing, a sample size of at least 5 is needed for the result to be significant. For the paired *t*-test there is no such limitation. Notably, after correction for multiple testing, almost all differences were no longer nearly significant (Table 2.1 through Table 2.5 and Figure 6).

**Table 2.1:** **Comparison of the median relative abundance corresponding to 6 bacterial species on phylum taxon present in all samples before (Mode A) and after (Mode B) supragingival cleansing: median and IQR, *p*-values for paired *t*-test and Wilcoxon signed-rank test and sample size calculation**.

**Taxon: Bacteriae**	**Sampling mode A (%)**			**Sampling mode B (%)**			**Paired *t*-test**	**Wilcoxon signed-rank test**		
**Phylum**	**25th**	**Median**	**75th**	**25th**	**Median**	**75th**	***p*-value**	***p*-value**	**Power**	**Sample size^§^**
Actinobacteria	5.4	8.2	17.9	5.5	11.7	22.6	0.637	0.813	0.012	264
Bacteroidetes	9.4	12.8	21.5	14.0	14.7	20.1	0.699	1.000	0.011	396
Firmicutes	27.4	34.5	44.2	20.5	26.0	47.5	0.708	0.813	0.011	423
Fusobacteria	4.0	4.9	6.7	4.0	6.5	14.6	0.311	0.813	0.030	54
Proteobacteria	17.1	22.1	43.9	13.1	22.8	35.6	0.488	0.813	0.017	120
Other_p1	3.2	3.8	5.7	3.0	4.9	5.5	0.980	1.000	0.008	91,086

§ *Power of 0.85 is assumed*.

*After Bonferroni correction p < 0.0083 is significant*.

**Table 2.2:** **Comparison of the median relative abundance corresponding to 14 bacterial species on class taxon present in all samples before (Mode A) and after (Mode B) supragingival cleansing: median and IQR, *p*-values for paired *t*-test and Wilcoxon signed-rank test and sample size calculation**.

**Taxon:Bacteriae**	**Sampling mode A (%)**			**Sampling mode B (%)**			**Paired *t*-test**	**Wilcoxon signed-rank test**		
**Phylum/Class**	**25th**	**Median**	**75th**	**25th**	**Median**	**75th**	***p*-value**	***p*-value**	**Power**	**Sample size^§^**
**ACTINOBACTERIA**										
Actinobacteria	5.4	8.2	17.9	5.5	11.7	22.6	0.637	0.813	0.005	304
**BACTEROIDETES**										
Bacteroidia	1.6	3.7	5.1	4.7	7.9	11.9	0.006	0.063	0.428	7
Flavobacteria	5.1	9.3	17.9	3.0	6.6	13.7	0.485	0.625	0.008	137
Other_c1	0.2	0.4	0.6	0.2	0.3	0.6	0.817	1.000	0.004	1278
**FIRMICUTES**										
Bacilli	25.3	31.1	38.0	16.1	23.2	42.6	0.673	0.813	0.005	381
Clostridia	1.7	26.5	5.0	2.1	3.5	5.0	0.617	0.438	0.006	271
Other_c2	0.4	0.8	1.2	0.5	0.6	1.0	0.774	1.000	0.004	831
**FUSOBACTERIA**										
Fusobacteria	4.0	4.9	6.7	4.0	6.5	14.6	0.311	0.813	0.014	63
**OTHER**										
Other_c3	3.2	3.8	5.7	3.0	4.9	5.5	0.980	1.000	0.004	105,044
**PROTEOBACTERIA**										
Alphaproteobacteria	0.1	0.4	1.1	0.2	0.5	0.6	0.714	0.813	0.005	507
Betaproteobacteria	6.7	14.0	26.7	7.2	12.4	27.1	0.991	1.000	0.004	555,484
Epsilonproteobacteria	0.3	1.3	2.4	0.8	1.3	3.7	0.587	1.000	0.006	229
Gammaproteobacteria	5.3	9.1	16.0	2.4	6.3	7.0	0.129	0.125	0.038	26
Other_c4	0.3	0.3	0.6	0.1	0.3	0.6	0.491	0.625	0.008	140

§ *Power of 0.85 is assumed*.

*After Bonferroni correction p < 0.0036 is significant*.

**Table 2.3:** **Comparison of the median relative abundance corresponding to 19 bacterial species on order taxon present in all samples before (Mode A) and after (Mode B) supragingival cleansing: median and IQR, *p*-values for paired *t*-test and Wilcoxon signed-rank test and sample size calculation**.

**Taxon: Bacteriae**	**Sampling mode A (%)**			**Sampling mode B (%)**			**Paired *t*-test**	**Wilcoxon signed-rank test**		
**Phylum/Order**	**25th**	**Median**	**75th**	**25th**	**Median**	**75th**	***p*-value**	***p*-value**	***p*ower**	**Sample size^§^**
**ACTINOBACTERIA**										
Actinomycetales	5.3	7.9	17.6	5.4	8.6	17.5	0.976	0.813	0.003	82,194
**BACTEROIDETES**										
Bacteroidales	1.6	3.7	5.1	4.7	7.9	11.9	0.006	0.063	0.353	8
Flavobacteriales	5.1	9.3	17.9	3.0	6.6	13.7	0.485	0.625	0.006	144
Other_o1	0.2	0.4	0.6	0.2	0.3	0.6	0.817	1.000	0.003	1344
**FIRMICUTES**										
Bacillales	1.6	3.5	4.2	2.0	2.7	9.0	0.220	0.313	0.015	44
Lactobacillales	20.7	24.4	32.8	12.5	17.9	31.7	0.487	0.625	0.006	145
Bacilli^*^	1.5	2.0	3.1	1.6	2.1	2.4	0.600	0.438	0.004	258
Clostridiales	1.7	2.6	5.0	2.1	3.5	5.0	0.623	0.438	0.004	294
Other_o2	0.4	0.8	1.2	0.5	0.6	1.0	0.774	1.000	0.003	874
**FUSOBACTERIA**										
Fusobacteriales	4.0	4.9	6.7	4.0	6.5	14.7	0.311	0.813	0.010	66
**PROTEOBACTERIA**										
Burkholderiales	0.3	4.7	9.8	0.7	1.6	12.6	0.800	1.000	0.003	1121
Neisseriales	3.0	13.6	16.7	4.5	10.9	14.9	0.834	0.813	0.003	1643
Betaproteobacteria^*^	0.2	0.4	1.0	0.0	0.2	1.5	0.663	1.000	0.004	376
Campylobacterales	0.3	1.3	2.4	0.8	1.3	3.7	0.585	1.000	0.004	238
Cardiobacteriales	0.8	1.2	3.7	0.3	1.7	2.6	0.615	0.625	0.004	282
Gammaproteobacteria^*^	0.2	0.3	0.8	0.2	0.3	0.6	0.528	0.813	0.005	177
Pasteurellales	1.7	5.5	14.8	1.3	3.9	4.3	0.246	0.313	0.014	49
Other_o3										
Other	0.3	0.3	0.6	0.1	0.3	0.6	0.491	0.625	0.006	147
Other_o4	3.2	3.8	5.7	3.0	4.9	5.5	0.980	1.000	0.003	110,445

* *Taxa marked with asterisk could not be assigned to any of the ordera and are shown on class level as lowest common taxon*.

§ *Power of 0.85 is assumed*.

*After Bonferroni correction p < 0.0026 is significant*.

**Table 2.4:** **Comparison of the median relative abundance corresponding to 27 bacterial species on family taxon present in all samples before (Mode A) and after (Mode B) supragingival cleansing: median and IQR, *p*-values for paired *t*-test and Wilcoxon signed-rank test and sample size calculation**.

**Taxon:Bacteriae**	**Sampling mode A (%)**			**Sampling mode B (%)**			**Paired *t*-test**	**Wilcoxon signed-rank test**		
**Phylum/Family**	**25th**	**Median**	**75th**	**25th**	**Median**	**75th**	***p*-value**	***p*-value**	**Power**	**Sample size^§^**
**ACTINOBACTERIA**										
Actinomycetaceae	0.9	1.4	3.9	1.4	2.5	4.3	0.572	0.438	0.003	232
Micrococcaceae	0.1	0.3	2.6	0.2	0.2	3.4	0.332	0.625	0.007	76
Actinomycetales^*^	3.4	5.3	9.9	1.3	2.7	12.9	0.965	0.625	0.002	38,446
**BACTEROIDETES**										
Porphyromonadaceae	1.1	1.5	3.5	1.2	3.1	5.4	0.183	0.313	0.014	38
Prevotellaceae	0.4	0.9	1.9	1.9	3.6	6.5	0.026	0.063	0.097	12
Flavobacteriaceae	5.1	9.3	17.9	3.0	6.6	13.7	0.485	0.625	0.004	150
Other_f1	0.2	0.4	0.6	0.2	0.3	0.6	0.817	1.000	0.002	1407
**FIRMICUTES**										
Bacillaceae	0.3	0.6	2.1	0.7	1.8	2.5	0.228	0.188	0.011	48
Staphylococcaceae	0.7	2.1	2.8	0.1	2.6	6.1	0.288	0.313	0.008	63
Carnobacteriaceae	1.6	2.2	3.1	0.4	0.5	2.2	0.061	0.125	0.045	18
Lactobacillales^*^	1.2	5.4	9.1	1.7	2.0	11.3	0.827	1.000	0.002	1575
Streptococcaceae	8.9	13.1	26.7	8.0	12.5	19.1	0.538	0.438	0.004	194
Bacilli^**^	1.5	2.0	3.1	1.6	2.1	2.4	0.600	0.438	0.003	270
Lachnospiraceae	0.6	1.3	3.1	0.3	2.4	2.7	0.951	0.813	0.002	20,474
Clostridiales^*^	0.8	1.0	2.0	1.2	1.6	1.8	0.737	0.813	0.002	665
Other_f2	0.4	0.8	1.2	0.5	0.6	1.0	0.774	1.000	0.002	915
**FUSOBACTERIA**										
Fusobacteriaceae	1.7	2.9	5.2	3.0	6.0	13.3	0.214	0.438	0.012	45
Leptotrichiaceae	1.3	2.2	2.3	0.6	0.7	1.6	0.122	0.188	0.004	143
**OTHER**										
Other_f3	3.2	3.8	5.7	3.0	4.9	5.5	0.980	1.000	0.002	115,633
**PROTEOBACTERIA**										
Burkholderiales^*^	0.3	4.7	9.7	0.7	1.5	12.5	0.797	1.000	0.002	1137
Neisseriaceae	3.0	13.6	16.7	4.5	10.9	14.9	0.834	0.813	0.002	1720
Betaproteobacteria^**^	0.2	0.4	1.0	0.0	0.2	1.5	0.663	1.000	0.003	394
Campylobacteraceae	0.3	1.3	2.4	0.8	1.3	3.7	0.581	1.000	0.003	244
Cardiobacteriaceae	0.8	1.2	3.7	0.3	1.7	2.6	0.615	0.625	0.003	295
Gammaproteobacteria^**^	0.2	0.3	0.8	0.2	0.3	0.6	0.528	0.813	0.004	186
Pasteurellaceae	1.7	5.5	14.7	1.3	3.8	4.3	0.246	0.313	0.010	52
Other_f4	0.3	0.3	0.6	0.1	0.3	0.6	0.491	0.625	0.004	154

* *Taxa marked with asterisk could not be assigned to any of the family taxon and are shown on ordera level as lowest common taxon*.

** *Taxa marked with asterisk could not be assigned to any of the family taxon and are shown on class level as lowest common taxon*.

§ *Power of 0.85 is assumed*.

*After Bonferroni correction p < 0.0019 is significant*.

**Table 2.5:** **Comparison of the median relative abundance corresponding to 29 bacterial species on genus taxon present in all samples before (Mode A) and after (Mode B) supragingival cleansing: median and IQR, *p*-values for paired *t*-test and Wilcoxon signed-rank test and sample size calculation**.

**Taxon:Bacteriae**	**Sampling mode A (%)**			**Sampling mode B (%)**			**Paired *t*-test**	**Wilcoxon signed-rank test**		
**Phylum/Genus**	**25th**	**Median**	**75th**	**25th**	**Median**	**75th**	***p*-value**	***p*-value**	**Power**	**Sample size^§^**
**ACTINOBACTERIA**										
Actinomyces	0.9	1.4	3.8	1.4	2.5	4.3	0.549	0.438	0.003	209
Rothia	0.1	0.3	2.5	0.1	0.2	3.3	0.333	0.625	0.006	77
Actinomycetales^**^	3.4	5.3	9.9	1.3	2.7	12.9	0.965	0.625	0.002	39,057
**BACTEROIDETES**										
Porphyromonadaceae^*^	0.0	0.1	0.6	0.1	0.2	0.6	0.555	0.438	0.003	215
Prevotellaceae^*^	0.1	0.3	0.4	0.2	0.7	1.2	0.116	0.063	0.021	27
Prevotella	0.3	0.5	1.5	1.0	3.3	5.7	0.047	0.063	0.051	16
Capnocytophaga	3.9	8.4	15.5	2.0	4.8	11.9	0.472	0.438	0.004	144
Flavobacteriaceae^*^	0.8	1.0	2.7	1.0	1.6	1.9	0.797	1.000	0.002	1153
Other_g1	0.2	0.4	0.6	0.2	0.3	0.6	0.817	1.000	0.002	1429
**FIRMICUTES**										
Gemella	0.7	2.1	2.8	0.1	2.6	6.1	0.288	0.313	0.007	64
Granulicatella	1.5	2.2	3.0	0.4	0.4	2.2	0.509	0.125	0.006	77
Lactobacillales^**^	1.2	5.4	9.1	1.7	2.0	11.3	0.827	1.000	0.002	1600
Streptococcus	8.9	13.1	26.7	8.0	12.5	19.1	0.537	0.438	0.003	197
Bacilli^***^	1.5	2.0	3.1	1.6	2.1	2.4	0.600	0.438	0.003	274
Lachnospiraceae^*^	0.6	1.3	3.0	0.3	2.3	2.6	0.856	1.000	0.002	2328
Clostridiales^**^	0.8	1.0	2.0	1.2	1.6	1.8	0.737	0.813	0.002	676
Other_g2	0.4	0.8	1.2	0.5	0.6	1.0	0.774	1.000	0.002	930
**FUSOBACTERIA**										
Fusobacterium	1.7	2.8	5.2	3.0	6.0	13.2	0.213	0.438	0.011	45
Leptotrichia	1.3	2.2	2.3	0.6	0.7	1.6	0.117	0.188	0.021	27
**OTHER**										
Other_g3	3.2	3.8	5.7	3.0	4.9	5.5	0.980	1.000	0.002	117,468
**PROTEOBACTERIA**										
Burkholderiales^**^	0.3	4.7	9.7	0.7	1.5	12.5	0.797	1.000	0.002	1155
Neisseria	0.2	0.7	8.8	0.5	0.9	6.9	0.836	1.000	0.002	1780
Neisseriaceae^*^	2.2	7.1	10.1	1.9	6.4	9.6	0.293	0.313	0.007	65
Betaproteobacteria^***^	0.2	0.4	1.0	0.0	0.2	1.5	0.663	1.000	0.002	400
Campylobacter	0.3	1.3	2.4	0.8	1.3	3.7	0.581	1.000	0.003	248
Cardiobacterium	0.8	1.2	3.6	0.3	1.5	2.5	0.601	0.625	0.003	276
Gammaproteobacteria^***^	0.2	0.3	0.8	0.2	0.3	0.6	0.528	0.813	0.003	188
Pasteurellaceae^*^	1.6	5.3	14.6	1.3	3.8	4.1	0.242	0.313	0.009	52
Other_g4	0.3	0.3	0.6	0.1	0.3	0.6	0.491	0.625	0.004	157

* *Taxa marked with asterisk could not be assigned to any genera and are shown on family level as lowest common taxon*.

** *Taxa marked with asterisk could not be assigned to any genera and are shown on order level as lowest common taxon*.

*** *Taxa marked with asterisk could not be assigned to any genera and are shown on class level as lowest common taxon*.

§ *Power of 0.85 is assumed*.

*After Bonferroni correction p < 0.0017 is significant*.

#### Sample size

Based on the Wilcoxon signed rank test and the assumption of a power of 85% (as required by the local Ethics Committee), a high variety of different sample sizes are thus needed for the bacterial representatives on different taxonomic levels. The bigger the effect size and the smaller the standard deviations, the fewer samples are needed. For example Table 2.3 shows that the calculated sample sizes needed for the 19 bacterial species on order level ranged between 8 for *Bacteroidales* (median *A* = 3.7; median *B* = 7.9) and 82,194 for *Actinomycetales* (median *A* = 7.9; median *B* = 8.6), despite the huge sample size of 110,445 needed for more or less rare and undefined representatives. At class level, only two more subjects for *Bacteroidetes* (phylum)-*Bacteroidia* and, at order level, only three more subjects for *Bacteroidetes* (phylum)-*Bacteroidales* would have been needed to reach a power of 85% and to obtain a significant result for the Wilcoxon signed rank test, assuming that effect size and standard deviation remain constant (see Table 2).

### Multivariate analysis: principal coordinate analysis (PCoA) and hierarchical clustering

**Figure 7:**
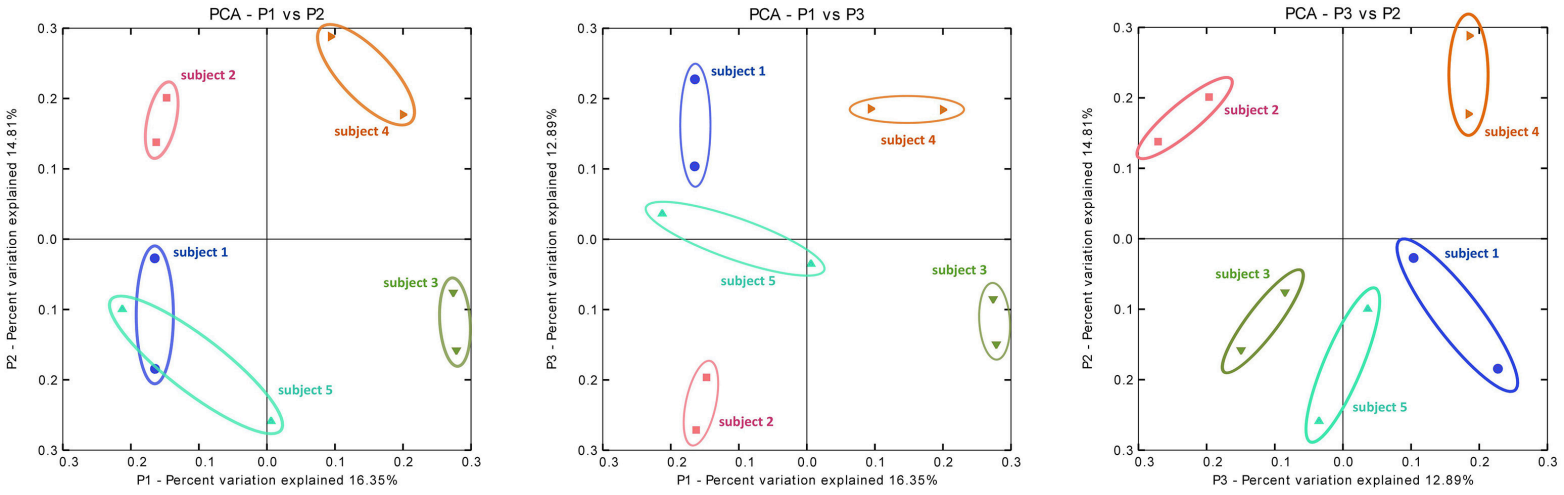
**Principal coordinate analysis (PCoA) on distance matrices calculated with unweighted UniFrac showing a grouping of the paired samples**. PCoA plots of the subgingival microbiome profiles of five healthy children based on two run-throughs of paper point sampling. Colors and symbols represent one child. The results using Adonis (Permutational MANOVA) did not reveal grouping of the samples according to Mode A or Mode B (*p* = 0.914 by *R*^2^ = 0.09).

**Figure 8:**
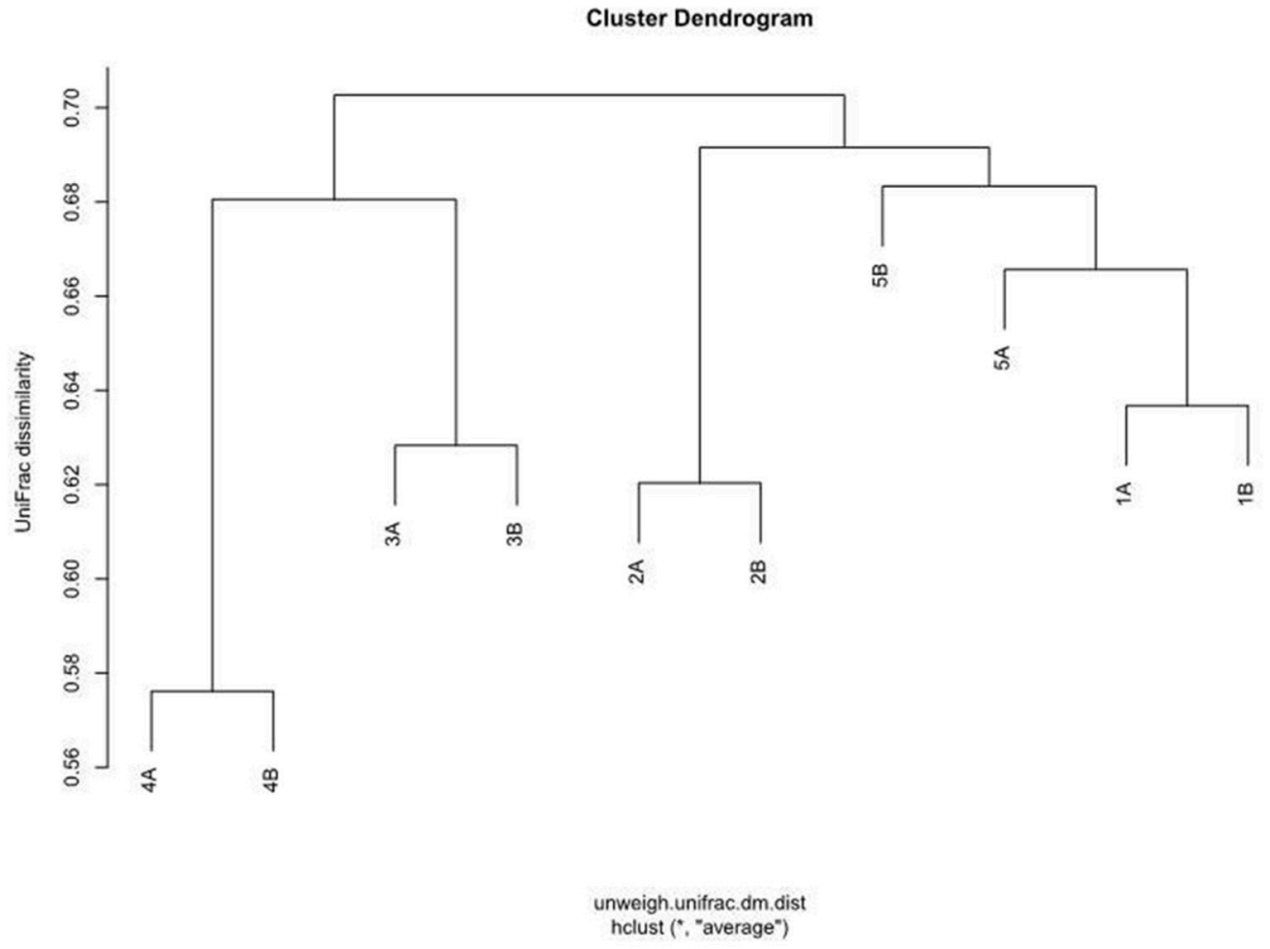
**Agglomerative hierarchical cluster analysis with average similarity based on unweighted UniFrac distances**. The data employed are the rarefaction based normalized OTU abundances of five healthy children based on two run-throughs of paper point sampling with slight modification of the clinical sampling technique.

Principal coordinate analysis (PCoA) on distance matrices calculated with unweighted UniFrac showed a grouping of the paired samples (Figure 7). Pairs, shown in the same color, are close together in all three dimensions, except for Sample 5A that shows a respective deviation and shows similarity with both samples from Subject 1. The results using Adonis (Permutational MANOVA) revealed no grouping of the samples according to Mode A or Mode B (*p* = 0.914 by *R*^2^ = 0.09) and thus no significant effects between Mode A and Mode B. PCoA results showed greater variability BETWEEN than WITHIN individuals. This observation was also supported by agglomerative hierarchical clustering analysis with average linkage on unweighted UniFrac distance (Figure 8).

## Discussion

Oral microbiota are considered one of the main risk factors for periodontal diseases affecting up to 90% of the world population (Pihlstrom et al., 2005). Oral biofilms have become increasingly important as a source of caries and periodontal disease as well as other bacterial infections in the human organism (Benítez-Páez et al., 2014). Some studies reveal evidence that oral pathogens play a role in various inflammatory diseases (Offenbacher et al., 2008). Few studies have deeply analyzed the composition of subgingival biofilm and elucidated the phylotypes/species associated with health or disease (Paster et al., 2001; Socransky and Haffajee, 2005; Ledder et al., 2007; Diaz, 2012; Abusleme et al., 2013).

The presented study analyzed using 454-pyrosequencing the data of five healthy 10-year-old children whose subgingival biofilm was examined excluding and including supragingival cleansing (Mode A and Mode B, respectively). The study aimed at assessing the effect of a slight modification of the clinical sampling technique for its accuracy in reflecting subgingival microbiome sequence data.

Retrieving adequate and reproducible samples is a challenge but awareness of the natural variability within subgingival microprints would enable us to distinguish pathological patterns at an early stage of disease. Corresponding *in vivo* conditions can best be studied in healthy children as shown in previous studies.

However, very few oral microbiome studies in healthy children have been performed so far (Papaioannou et al., 2009; Xin et al., 2013), some including pyrosequencing (Crielaard et al., 2011; Stahringer et al., 2012; Ling et al., 2013; Lif Holgerson et al., 2015). The study design of Crielaard et al. differs from ours in that they investigated microbial profiles of saliva collected from caries-diseased Dutch children aged 3–18 years. The biggest difference in the comparable age strata was the relative abundance of *Firmicutes* at 58% in the saliva group and at 30% in our subgingival samples, while the latter presented a higher proportion of *Proteobacteria* (22 vs. 12%) and *Fusobacteria* (6 vs. 2%). Ling et al. used parallel barcoded 454-pyrosequencing to study the diversity and richness of salivary bacteria in 10 healthy children and adults. The bacterial diversity was found to be more complex in children than in adults (Ling et al., 2013) which could be interpreted as evidence for the relationship between biodiversity and health. In their sample comprising 60 children aged 3–6, the eight predominant phyla in supragingival plaque and saliva were present in proportions that were comparable to our study: 23–42% *Firmicutes* and 16–37% *Bacteroides* (Ling et al., 2010). In a longitudinal study, Holgerson et al. looked at the oral microbiota of 207 Swedish babies at the age of 3 months and again at 3 years. The pyrosequencing data referred to 11 children with and 11 without caries. A significant increase in species richness and taxa diversity was described. Several taxa within the oral biofilms of the 3-year-olds could be linked to the presence or absence of caries. However, quantitative comparisons of the oral microbiota of children are possible only to a limited extent, since the investigators dedicated work differs in parameters such as study population (age, country, caries status), sampling sites (saliva, mucosal, and supragingival plaque) and molecular methods (DNA-DNA checkerboard, micro arrays, pyrosequencing). Papaioannou et al., for example, looked at five different oral habitats (saliva, tongue, soft tissue, subgingival, and total supragingival plaque) of 93 children from three different age groups using whole genomic probes for 38 species and the checkerboard DNA-DNA hybridization technique. The authors suggest a gradual maturation of the oral microbiota in children displaying patterns of colonization similar to those seen in adults (Papaioannou et al., 2009). However, until now most studies have analyzed salivary biofilm, as it is easier to sample (Luo et al., 2012; Stahringer et al., 2012; Ling et al., 2013; Gomar-Vercher et al., 2014). Interestingly, Luo et al. who studied PCR-amplified bacterial DNA from the saliva of 20 children with caries and of 30 healthy ones found higher microbial diversity in samples from diseased oral cavities. In contrast to these findings, Gomar-Vercher et al. used pyrosequencing to analyze 110 saliva samples from children split into six groups according to caries severity and found the bacterial diversity to decrease with progressing disease. At the same time, intra-group differences were considerable (Gomar-Vercher et al., 2014). Stahringer and colleagues presented a longitudinal survey of salivary microbiota from twins using PCR amplification and 454 pyrosequencing of the 16S rDNA hypervariable regions V1 and V2. Their findings point to the environment as the microbiome-determining factor showing greater differences between non-related subjects than within individuals or between twins (as long as they share a common habitat; Stahringer et al., 2012).

Standardized sampling procedures are a prerequisite for comparing subgingival microbiome data derived from research worldwide. The lack of heterogeneity and standardization for clinical protocols poses a limitation to data quality which should be noted by clinicians and microbiologists. In this context, we need to consider the diverse sampling methods reported for the collection of samples from a healthy oral cavity, not to mention the variability of pocket sampling in periodontally diseased patients. This can be illustrated by the example of just 10 published manuscripts dealing with the collection of samples from an intact oral cavity. They report using saline oral wash rinse (Ahn et al., 2011) or unstimulated whole saliva (Xin et al., 2013) for fluid collection; dental explorers (Xin et al., 2013), metal loops (Ling et al., 2010), metal curettes (Papaioannou et al., 2009) and wooden tooth picks (Keijser et al., 2008) for supragingival sampling; or wet and dry swabs and brushes (Aas et al., 2005; Papaioannou et al., 2009; Cortelli et al., 2012) and spatulas (Gohler et al., 2014) for mucosal sampling. Finally, subgingival sampling is currently being performed using either metal curettes (Papaioannou et al., 2009; Abusleme et al., 2013) or paper points (Cortelli et al., 2012; Griffen et al., 2012; Jünemann et al., 2012). For clinical and research purposes even exotic micropipettes or microelectrodes are used (Geibel, 2006). Potential sampling variability springs not only from the different instruments that can be utilized but also from processes taking place prior to sampling, such as plaque control, tooth cleaning, tooth isolation and drying, as well as from inadequate specifications regarding the sampling technique and time lines. Compared to standards that apply in other medical and laboratory settings, our clinical sampling is much like an elephant in a porcelain shop. Appropriate scientific input facilitates the development of a systematic and precise methodology which in turn can deliver reliable, high-quality clinical samples to the pipeline required in the field of molecular biology and medicine. Some authors have reported on the recovery of putative pathogens from paper point and curette sampling (Jervøe-Storm et al., 2007; Teles et al., 2008; Angelov et al., 2009; Sahl et al., 2014). Hartroth and colleagues have evaluated paper point sampling on bench (Hartroth et al., 1999), but these findings have yet to be tested under clinical conditions to establish the best practice. An aspect on which clinical researchers are in agreement is the removal of supragingival plaque before subgingival sampling. It is as obvious to them as taking off the shoes in the hallway before entering the living room. However, it is still debatable to what extent this cleansing should be performed to be efficient enough.

Generally, clinical sampling within the oral cavity of children can be tricky and calls for an experienced investigator. The clinical method in this study is designed around a younger study population with intact and tight subgingival compartments. The subgingival sulcus itself can best be imagined as an interface (of two millimeters) with a tight epithelium toward the periodontium but with a seamless junction (orifice) toward the supragingival surface. Thus, not only the removal of non-attached bacteria but also the microbial exchange between sub- and supragingival biofilm has to be taken into account in addition to the difficulty of precise sampling in this extremely limited subgingival space. Limited space makes sampling the subgingival sulcus of children a challenge. The deeper the sulcus, the more likely it is to strip supragingival biofilm before actually reaching the sulcus depth. In our case, sampling was performed by a single, experienced clinician excluding interrater variability. Paper points were gently slid parallel to the gingival margin in order to facilitate a painless and quick examination. This contributes to better cooperation on behalf of the child and a short procedure prevents the paper point from becoming saturated with saliva. Paper points were used rather than the more invasive metal curette, as the latter could traumatize the subgingival sulcus and cause bleeding which was to be avoided at all costs. During the sampling procedure, the focus was placed on the drier subgingival areas of the upper arch, so as to optimize sample quality for DNA analysis. To ensure reproducibility, biofilm sampling followed a strict protocol (see also Methods above). Two modes (A and B) of the same sampling method were used for comparison. After supragingival cleaning using an electric toothbrush and water, sampling was performed, firstly, excluding (Mode A) and, secondly, including (Mode B) cleansing with sterile cotton pellets. The samples from a total of eight sites were pooled, so no inter-site comparisons were studied. Based on the paired samples, results of the PCoA intra-individual differences were relatively small despite the modest sample size in the present study. Also, permutational MANOVA showed no grouping of the samples according to Mode A or Mode B. It can be speculated that any existing deviation between the two sampling modes is very likely to correspond to a natural variation in oral biofilm of the individual subject and supragingival cleansing with a sterile cotton swab does not affect the composition of the subgingival biofilm of an individual. Importantly, it seems that there are no major effects due to the described sampling modification. However these “non-effects” between the two sampling modes refer to inter-individual differences and obviously surpass the intra-individual “non-effects” which comes up to an overarching effect with relevance for future clinical studies.

Analyses of the pooled DNA data using pyrosequencing is a timely and potentially interesting approach that also has numerous limitations. Table 1 shows that richness and evenness as well as Shannon diversity index do not indicate any differences in the above mentioned sampling modes (A and B). However, it should be noted that the number of reads can influence the sensitivity of data; this issue is for example evident when comparing the quotient of the number of reads and richness for subjects 4A and 5A. Such differences in the number of reads are practically unavoidable, therefore it is necessary to incorporate a normalization step into the data analysis which we did by rarefication of all samples to the sample with the lowest number of reads. Another option is the use of relative abundances as also applied in this study for statistical comparisons. The field under study here is so complex that it is impossible to ascertain at which exact point in the analysis problems occur, and whether the same amount of DNA was available from the participating children and/or if data loss had occurred even earlier. Laboratory workup is not discussed here in detail but the possibility of passive errors (e.g., during 16S RNA amplification for PCR) does exist despite standardized procedures. It also has to be remembered that this study looked at 16S rRNA hypervariable regions V5 and V6 only and not at the whole metagenome. This limitation also applies to other studies (Wu et al., 2010; Ahn et al., 2011; Griffen et al., 2011; Jünemann et al., 2012; Stahringer et al., 2012).

An asset of our study is the fact that (under the afore-mentioned conditions and for the afore-mentioned subjects) sample size calculations are presented for the bacterial species on all five levels as shown in Tables 2.1–2.5. Our small sample size poses a challenge for pyrosequencing and statistical testing, nevertheless different effects can be observed as visualized in Figures 6–8. In our study, sample sizes are part of the findings. Our work should emphasize that the challenge is the translation of sample size estimations to clinical feasibility. So far, statistically given sample sizes that would explain significantly and clinically relevant differences in the subgingival microbiome of children are neither practical nor ethical. Even a generous increase in samples, i.e. children, in our study would not have solved the problem. However, our data can serve as a pilot for future studies on the topic showing that large sample sizes are needed to elucidate microbial structures at different levels. The demanding task is to reflect the bacterial diversity as well as possible. However, as opposed to more common bacteria, rare species require huge sample sizes in order to unveil any significant differences. This task becomes even more complex with a higher number of rare species in a given sample. In order to study these issues, statistical methodology will have to be developed further. While appropriate technology is becoming increasingly available and affordable, sample sizes remain primarily a matter of practicality and ethics. Including healthy people, in particular children, or patients into clinical studies involves substantial costs for human resources and efforts beyond the daily routine for both sides: the study participants and the clinical staff. One way to practically increase sample sizes are standardized clinical protocols that would allow multi-site sampling in diverse populations.

In our analysis, the bacteria are only analyzed down to the genus level which is limiting. However, from the clinical perspective the data is noteworthy. Interestingly, the smallest calculated sample size roughly corresponds to the 20 bacteria available in commercial bacteria test kits applied in periodontology. However, some abundant bacteria are apparently not included in such test kits. In addition, numerous bacteria have not yet been identified and are assigned as “other” to superordinate taxonomic levels (see Tables 2.1–2.5). In this context, the limitation is the unattainable sample size for some phyla.

Another general issue that should be mentioned is the need for standardized protocols to facilitate the comparability of data generated in microbiome studies. Considerable inter-individual differences in bacterial communities necessitate large samples. At the same time, intra-individual variability should also be considered in comparative studies. For microbiome data, new statistical methods like Adonis are needed and should be combined with methods from bioinformatics. For example, PCoA was used in our study to verify findings based on a small sample size, i.e., grouping of the paired samples for the within-comparison as intra-individual pairs clustered in all three dimensions. Importantly, many decisions regarding study design are made based on investigator experience (e.g., which distances to analyze with UniFrac). Future studies should aim at standardizing methodology to prevent bias and distortion of data.

Our work points at many challenges in the study of oral microbiomes. Our data, though based on a modest sample size, could serve as a reference for healthy children or may serve as a baseline for microbiome function in healthy individuals shedding new light on the frontiers of health and disease. The number of the species known is high (presently amounting to more than 600 taxa) and includes very rare ones whose role is yet unknown as well as other microbial representatives that are not bacteria (Moissl et al., 2002, 2003, 2005). Methods like DNA/RNA/metagenome sequencing need to be employed to begin to uncover the exact role of microbiota. Similarly, visualized analytics can give additional insight into individual species. However, we still need to learn which microbiota are imperative for the functioning of the whole. And we need to ask further questions: How does diversity make healthy? To what extent may individual health be attained by comparison with other individuals? The presented work employs modern approaches from several research areas but the focus remains on the clinical application and a contribution toward the standardization of procedures across all relevant disciplines.

## Author contributions

ES: work conception and design; acquisition, analysis and interpretation of data; drafting and critical review of the manuscript; final approval of the work for publication. ST: analysis and interpretation of data; critical review of the manuscript; final approval of the work for publication. KE: analysis and interpretation of data; critical review of the manuscript; final approval of the work for publication. BK: study design; analysis and interpretation of data; draft and critical review of the manuscript; final approval of the work for publication.

## Funding

This study was supported by the Hygiene Fund of the Institute of Hygiene, Microbiology and Environmental Medicine at the Medical University of Graz, Graz, Austria.

### Conflict of interest statement

The authors declare that the research was conducted in the absence of any commercial or financial relationships that could be construed as a potential conflict of interest.

## Supplementary material

Supplementary Figure S1 **Ten rarefaction curves generated from subgingival microbiome profiles of five healthy children based on two performances of paper point sampling before (Mode A) and after (Mode B) supragingival cleansing with a sterile cotton pellet (colors designate sampling modes)**.

Supplementary Table S1 **Barcoded primer sequences used in this study: PCR ampicons were sequenced from the Titanium A adaptor (CCATCTCATCCCTGCGTGTCTCCGAC), followed by a 4 bases key sequence (TCAG) and the barcode**. The reverse primer was used with the Titanium B adaptor (CCTATCCCCTGTGTGCCTTGGCAGTC), the key sequence and the target specific sequence but without barcode sequence.
