# Supplementary figures and images for: Sampling Modification Effects in the Subgingival Microbiome Profile of Healthy Children
Source: Front Microbiol. 2017 Jan 18;7:2142. doi: 10.3389/fmicb.2016.02142 (PMC5241288; doi:10.3389/fmicb.2016.02142)

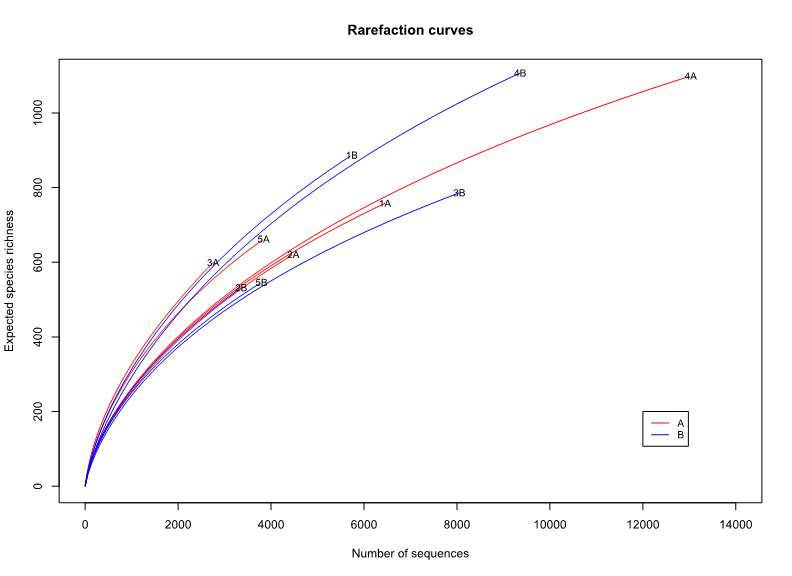

Supplement: Supplementary Figure S1 — Ten rarefaction curves generated from subgingival microbiome profiles of five healthy children based on two performances of paper point sampling before (Mode A) and after (Mode B) supragingival cleansing with a sterile cotton pellet (colors designate sampling modes). [file Image1.JPEG]
