# Supplementary material for: Sampling Modification Effects in the Subgingival Microbiome Profile of Healthy Children
Source: Front Microbiol. 2017 Jan 18;7:2142. doi: 10.3389/fmicb.2016.02142 (PMC5241288; doi:10.3389/fmicb.2016.02142)
Supplement: Supplementary Table S1 — Barcoded primer sequences used in this study: PCR ampicons were sequenced from the Titanium A adaptor (CCATCTCATCCCTGCGTGTCTCCGAC), followed by a 4 bases key sequence (TCAG) and the barcode. The reverse primer was used with the Titanium B adaptor (CCTATCCCCTGTGTGCCTTGGCAGTC), the key sequence and the target specific sequence but without barcode sequence. [file Table1.DOCX]

**Supplementary table S1:** Barcoded primer sequences used in this study: PCR amplicons were sequenced from the Titanium A adaptor (**CCATCTCATCCCTGCGTGTCTCCGAC**), followed by a 4 bases key sequence (TCAG) and the barcode. The reverse primer was used with the Titanium B adaptor (**CCTATCCCCTGTGTGCCTTGGCAGTC**), the key sequence and the target specific sequence but without barcode sequence.

Barcode ID MID sequ. (5‘ - 3‘) target specific sequence

1 ACGCTCGACA *AGGATTAGATACCCTGGTA*

2 AGACGCACTC *AGGATTAGATACCCTGGTA*

3 AGCACTGTAG *AGGATTAGATACCCTGGTA*

4 ATCAGACAC *AGGATTAGATACCCTGGTA*

5 ATATCGCGA *AGGATTAGATACCCTGGTA*

6 CGTGTCTC *AGGATTAGATACCCTGGTA*

7 TAGTAT *AGGATTAGATACCCTGGTA*

8 TGATA *AGGATTAGATACCCTGGTA*

9 AGACTATACT *AGGATTAGATACCCTGGTA*

10 CGACGTGACT *AGGATTAGATACCCTGGTA*

R no MID *CRRCACGAGCTGACGAC*
